# The Validation of Peer Review through Research Impact Measures and the Implications for Funding Strategies

**DOI:** 10.1371/journal.pone.0106474

**Published:** 2014-09-03

**Authors:** Stephen A. Gallo, Afton S. Carpenter, David Irwin, Caitlin D. McPartland, Joseph Travis, Sofie Reynders, Lisa A. Thompson, Scott R. Glisson

**Affiliations:** 1 American Institute of Biological Sciences – Scientific Peer Advisory and Review Services Division, Reston, Virginia, United States of America; 2 Florida State University, Department of Biological Science, Tallahassee, Florida, United States of America; Max Planck Society, Germany

## Abstract

There is a paucity of data in the literature concerning the validation of the grant application peer review process, which is used to help direct billions of dollars in research funds. Ultimately, this validation will hinge upon empirical data relating the output of funded projects to the predictions implicit in the overall scientific merit scores from the peer review of submitted applications. In an effort to address this need, the American Institute of Biological Sciences (AIBS) conducted a retrospective analysis of peer review data of 2,063 applications submitted to a particular research program and the bibliometric output of the resultant 227 funded projects over an 8-year period. Peer review scores associated with applications were found to be moderately correlated with the total time-adjusted citation output of funded projects, although a high degree of variability existed in the data. Analysis over time revealed that as average annual scores of all applications (both funded and unfunded) submitted to this program improved with time, the average annual citation output per application increased. Citation impact did not correlate with the amount of funds awarded per application or with the total annual programmatic budget. However, the number of funded applications per year was found to correlate well with total annual citation impact, suggesting that improving funding success rates by reducing the size of awards may be an efficient strategy to optimize the scientific impact of research program portfolios. This strategy must be weighed against the need for a balanced research portfolio and the inherent high costs of some areas of research. The relationship observed between peer review scores and bibliometric output lays the groundwork for establishing a model system for future prospective testing of the validity of peer review formats and procedures.

## Introduction

Some form of peer review is used at the majority of research granting organizations to determine the most meritorious applications to consider for funding. As such, peer review makes a significant contribution to how billions of dollars in research grants are awarded, influencing the very direction of science itself. However, this process has been increasingly questioned, particularly with regard to how well peer review results predict the ultimate impact of the funded research [1]–[5]. While several studies suggest that the process of peer review of scientific manuscripts has some success in identifying what will later become highly cited, high-impact publications, only a handful of publications have dealt with the predictive accuracy of the outcomes of peer review of grant applications [6]–[9]. Of these, a few have reported results supporting the validity of the peer review outcome [10]–[13]. However, in terms of direct comparison between peer review scores (or percentile ranking) and bibliometric data, several publications from program directors at the NIGMS and NHLBI have indicated either a modest (but statistically significant) correlation or no correlation between publication impact and peer review scores, with both data sets displaying a substantial amount of variation in impact among grants with similar peer review scores [14], [15].

Thus, the few studies in the literature that do exist provide inconsistent results at best and contradictory results at worst. In addition, the sources of the large degree of variability in the data from these studies remain unexplored, as has the dynamic relationship of publication impact and peer review output of a funding program over time. Understanding the factors that influence the inputs and outputs of funded research programs is crucial for two reasons. First, the results of such analyses can be used to develop a working model of the peer review process with which to validate evaluation procedures. Second, the results could inform funding agencies on how to optimize their funding strategies to promote the maximal scientific impact of their programs.

The American Institute of Biological Sciences (AIBS) has conducted a retrospective analysis of peer review and project output data over an 8-year period for a discrete funding research program and examined whether correlations exist among the assessment of scientific merit using a peer review system and the scientific output from this program.

## Background

**Figure 1:**
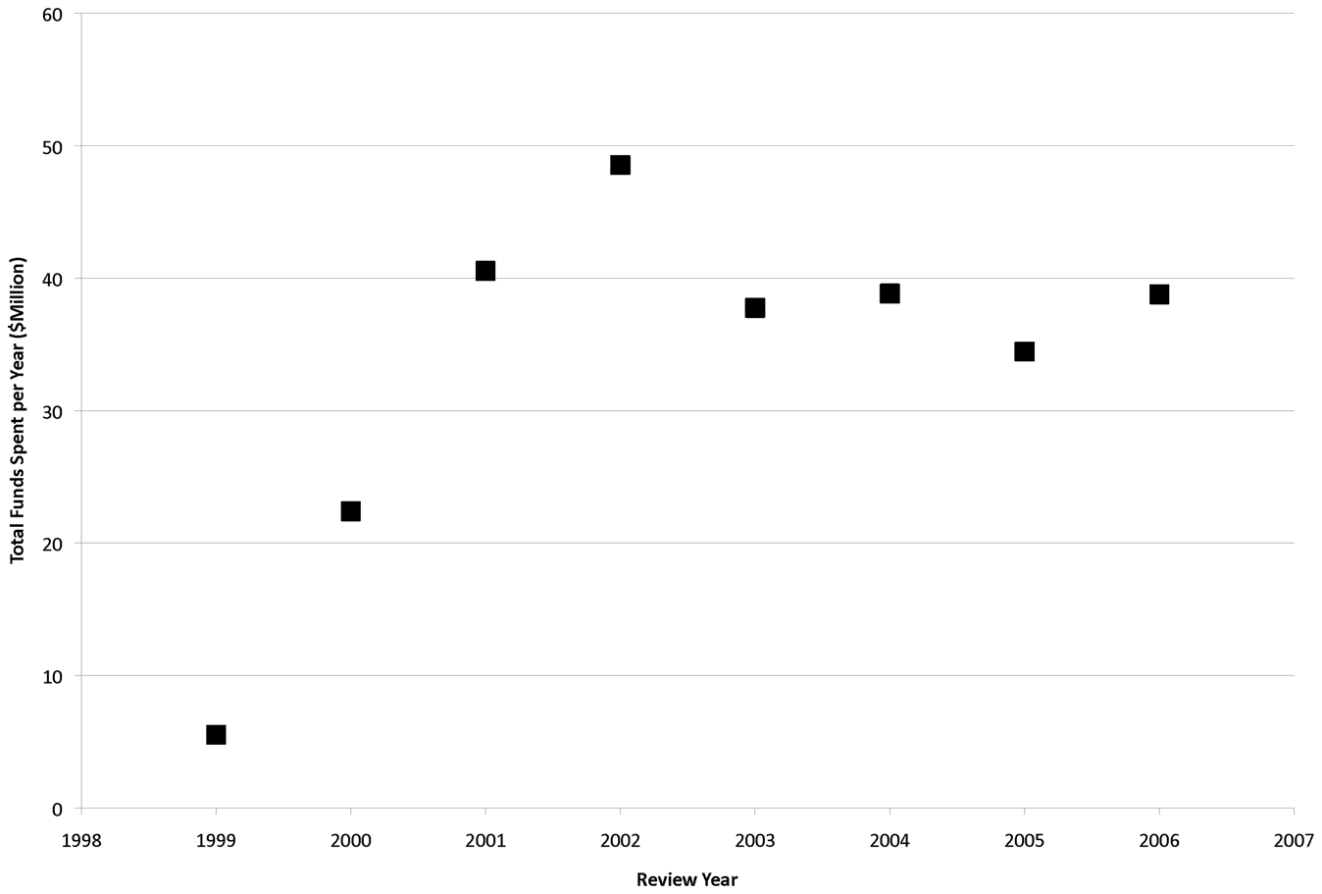
PrX Funding Levels over Time. Total funds spent per annual competition (in $Million) are plotted against time (1999–2006, review year).

AIBS conducts scientific peer review for federal and non-federal clients and in doing so has accumulated data that speak to the predictive ability of the peer review process. For one such program, referred to as PrX in this manuscript, AIBS has collected peer review scoring data and post-funding citation output data from applications reviewed between 1999 and 2006. PrX is an extramural program designed to support a wide variety of research topic areas, including vision, drug abuse, nutrition, blood-related cancer, kidney disease, autoimmune diseases, malaria, tuberculosis, osteoporosis, arthritis, and autism research, among others. Topic areas were not static, changing from year to year in both type and number (14–31 distinct areas per year), and very few were continuous throughout the 1999–2006 period of study. However, after an initial rise, the total number of topic areas did stabilize after 2001 to an average level of 27. In every program year there was a significant proportion of both applied and basic research applications, with many applications encompassing varying degrees of both basic and applied research in their specific aims. However, topic area descriptions were general and brief, with research scopes largely open to interpretation by the applicants (e.g., one such topic area was “Drug Abuse”; no further definition was provided). In general, the research submitted was overwhelmingly biomedical in nature over the full review period (1999–2006). The program began in 1999 with a funding level of $5.5 M, which increased to $40.5 M in 2001 and remained roughly at that level through 2006 (**Figure 1**). While this program has utilized several funding mechanisms, applications included in this analysis were submitted to a 4-year, R01-style support mechanism.

Data were collected from the peer review of 2,063 submitted applications encompassing an 8-year period from 1999 to 2006. The numbers of submitted applications were 91, 162, 182, 124, 247, 275, 432, and 550 for 1999–2006, respectively. PrX does not use a resubmission mechanism, so all applications are reviewed as new submissions by freshly convened panels of reviewers. Overall, 90% of the PrX applications were reviewed by on-site panels comprising, on average, 10 reviewers. Each panel evaluated on average 25 applications per panel over a two-day period. The remaining 10% of applications were reviewed individually by 3 reviewers and discussed via teleconference (all 91 applications in 1999 were reviewed this way, while in all subsequent other years on-site panel review was employed for the vast majority of applications). All reviewers were recruited for panels based on the expertise required for the applications submitted that year and 80–90% of the reviewers were new each year (i.e. no standing panels were used for this program).

The number of awards made per year increased with time (16, 15, 34, 30, 25, 26, 35, and 46 awards for 1999 to 2006, respectively). The median budget per funded application increased with time initially, and then dropped sharply in 2005 ($344K, $1493K, $1153K, $1575K, $1510K, $1493K, $985K, and $863K for 1999–2006, respectively). The relative proportion of junior-level, mid-level and senior-level funded investigators was 29%, 23%, and 48% respectively. The overall success rate decreased over time, from 18% in 1999 to 8% in 2006; this decrease was largely attributable to the increase in numbers of submitted applications. It should be noted that the success rate was calculated internally and not made available to the public, so applicants and reviewers were not aware of funding rates.

A two-tiered process was used: AIBS managed the first tier to evaluate scientific merit and supplied the funding agency with global priority scores and critiques for each submission. Then the funding agency convened an internal panel to make the final funding decisions based largely on the application critiques and scores, as well as programmatic relevance and balance of topic areas.

## Methods

Scoring data were recorded after the discussion of each application. Applications were scored on a scale of 1.0 to 5.0 with 0.1 increments (1.0 being the best score). The overall scientific merit score of each application was calculated as the average of scores from all panel members without a conflict. A description of the peer review procedures of this program is provided in the methods section below and further detail can be found in a recent publication [16].

The publication data related to each individual funded project (227 funded projects in total) were gleaned from the mandatory final reports submitted by the applicant to the funding agency. On average, these reports were submitted 5 years after the peer review of the project occurred. Therefore the reports covered the period from 2004 (generated from the 1999 review) to 2011 (from the 2006 review), with resultant publications produced from 1 to 8 (average 4.3) years after the review date. It should be noted that the final reports from funded grants (and the resultant publication lists) are a matter of public record and can be accessed by anyone. However, these reports reveal the name of the funding agency. AIBS is bound by contractual confidentiality clauses and, as such, cannot reveal the names of the applicants or the funding agency in this manuscript. We have, however, anonymized the data used for this analysis and provide it as part of this manuscript as (**File S1**).

In this analysis, we counted only peer-reviewed publications (confirmed through PubMed and Web of Knowledge searches) listed by the applicant in the final report. This means that we did not count meeting abstracts, technical reports, or similar types of products. We confirmed all listed publications via searches in PubMed and Web of Knowledge (Thomson Reuters). We also did not include deliverables such as patents, devices, and internal white papers and reports. If a publication was listed in the final report as “submitted” or “in preparation”, we conducted an Internet search for that title; if we found it, we included it in the count. We also tallied publication citation levels (as of 2014) through the use of Web of Knowledge.

We used a total of 805 peer reviewed publications and the resulting 20,313 citations for this analysis. The total citation level per funded project represents the cumulative citations of all publications resulting from the funded project. However, because citation level is a time-dependent quantity, all citation levels were standardized based on the average citation level of all publications in a field of science cited in each relevant year, as gleaned from a published calculation using the Thomson Reuters Essential Science Indicators database [17]. These published average rates were determined for 2000–2010 by scientific field, assessed in 2011 and displayed a linear relationship with time (e.g., R^2^ = 0.99 for the field of molecular biology). We chose molecular biology because it was the highest cited field and in general was the field most applicable to funded Prx applications. We did not standardize for research field in this analysis as most of the output had multiple dimensions (e.g., molecular biology and biochemistry) and ascribing one specific topic area to any publication would introduce error into the standardization process.

Because the Reuters curve was assessed in 2011, we extended the Reuters curve for 2014, back calculating through the use of the linear fit. In this way, we could most accurately standardize the PrX data for the relationship between publication date and citation level and could calculate the Total Relative Citation (TRC) level per application, which was used as a metric for impact to compare projects funded in different fiscal years.

## Results

### Review Scores versus Citation Impact

**Figure 2:**
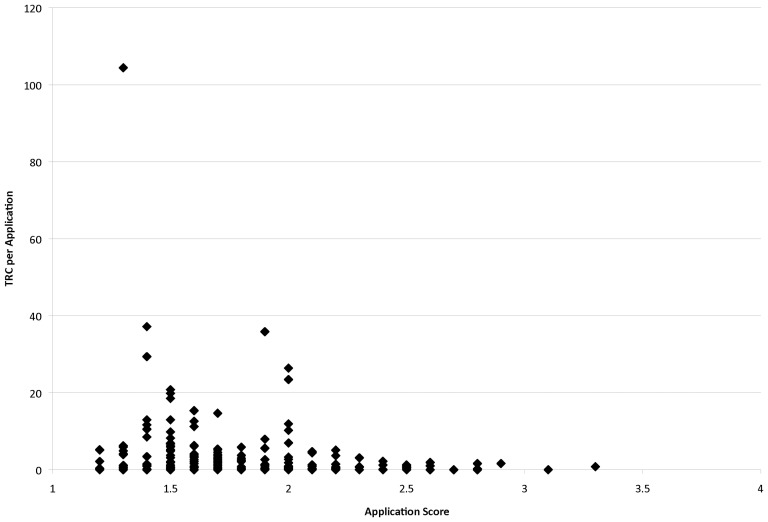
Total Relative Citation (TRC) per Application Versus Score. Summation of all citations from individual grant publication yield (normalized against Reuters average) from each funded PrX application was calculated. These values, referred to as average Total Relative Citations (TRC) were plotted versus individual peer review score (1999–2006, n = 227).

Scatter plots of peer review scores and TRC values showed a great deal of variability in citation output for applications of similar merit scores, particularly among the higher average scores (**Figure 2**). One prominent outlier (score of 1.3, TRC value of 150) was identified in this data set and removed to avoid the misinterpretation of a false trend of TRC with merit score. In a simple check for statistical significance, these raw data were separated into strong and weak scores using the 1.8 median funded application score as a threshold (1.7 and lower being the stronger applications and 1.8 or higher being the weaker applications). A two-tailed t-test of unequal variances of this grouping indicated a statistically significant difference between high and low scores for TRC levels (t[218] = 2.66; p = 0.008), despite the variability in citation output. This result also demonstrates the discriminant validity of the peer review scores.

**Figure 3:**
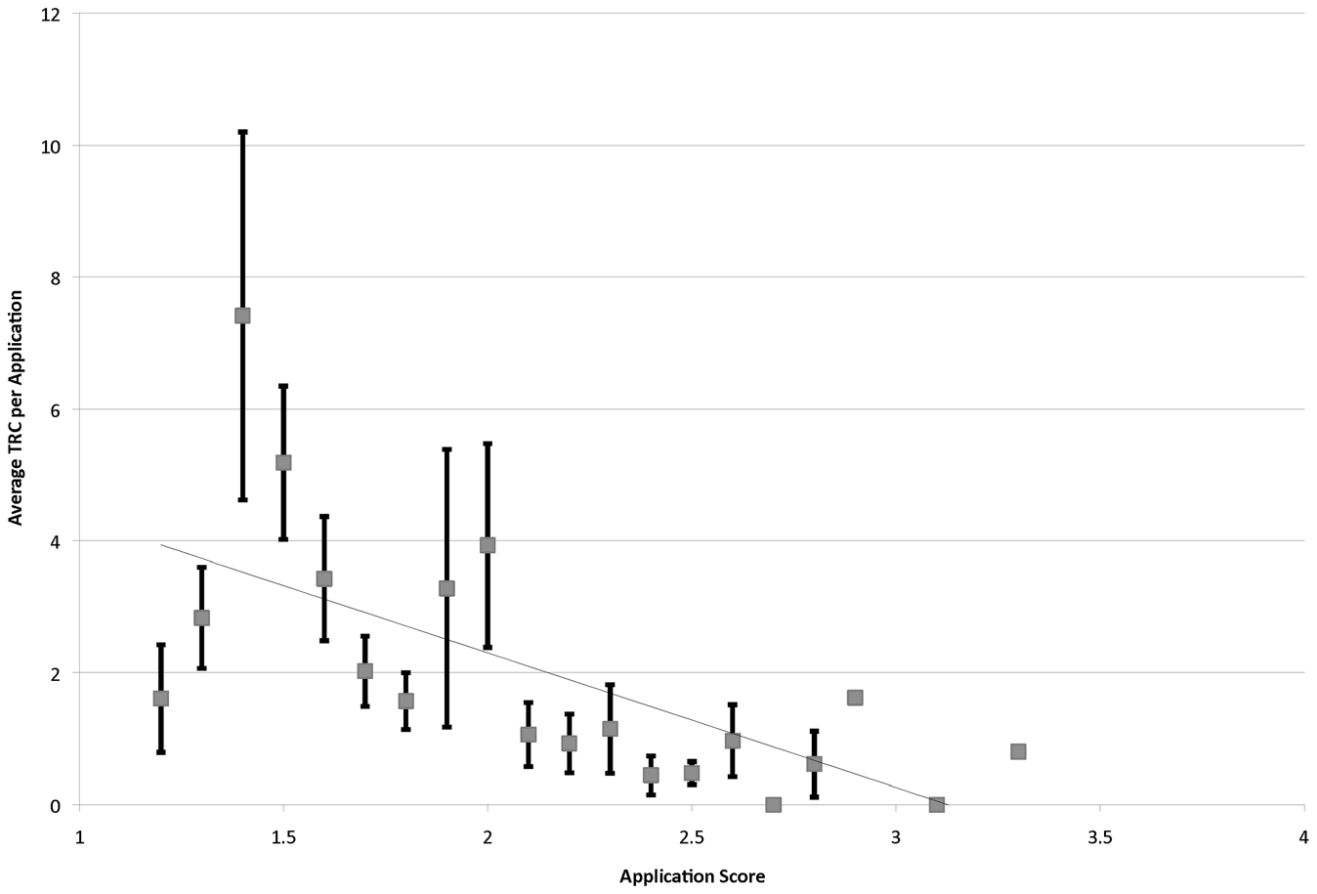
Average TRC Level versus Average Application Score Using Score Grouping. TRC was calculated for individual funded applications (1999–2006). Applications were then grouped by identical review score and then averaged. These average TRC values were plotted for the 21 scoring groups (n ranges from 1 to 30, depending on group) and fit with a linear function. Error bars represent the standard error of the mean.

We applied a regression analysis to the data by grouping proposals by their common average and using the common averages as predictors. This analysis shows a more consistent, continuous relationship with application score. Better-scoring applications on average produced a higher TRC level (**Figure 3**), yielding a linear fit with a slope of −2.04±0.54 (p = 0.001) and with a moderate coefficient of determination of R^2^  = 0.44 (p = 0.001). This relationship may not be monotonic, as a lack-of-fit test suggests that this linear model does not account for curvature in the data (t = 14.42; p = 0.001). Also, there was still considerable variability in this plot, and the variance of TRC increased greatly for better scoring applications (**Figure S1**). Nevertheless it seems, as in the NIGMS study, there was a moderate level of correlation between citation impact metrics and peer review score.

We also grouped the TRC counts per application by funding level, creating nine levels of funding in $400K increments. When we did this, we found no statistically significant difference amongst applications in TRC (F[8,217] = 1.50; p = 0.16). We also used the median funding level of $1M to demarcate two groups, “low” and “high” budgets, and yet found no effect of funding level on TRC (t[216] = 0.65; p = 0.52). This is consistent with others' findings [18], [19]. In addition, a poor correlation was found between budget and peer review score (R^2^ = 0.03, p = 0.65). Thus budget did not significantly contribute to the variability in the observed TRC data.

To explore the potential influence of investigator seniority and academic status as a covariate for TRC, we placed each investigator into one of three categories of academic rank: Junior, Mid-level or Senior academic rank groups (we did not include investigators whose titles were ambiguous, **Figure S2**). Analysis of variance by rank indicated no effect of rank on TRC output (F[2,188] = 0.41; p = 0.67).

### Time Analysis

While the data in this analysis are inclusive of all 8 funding years, and several factors have changed from year to year, including the PrX funding rate and overall increasing competition for funding, there is some correlation between scoring and TRC. To further explore these relationships, averaged peer review scores and levels of citation from the PrX program over time were examined.

**Figure 4:**
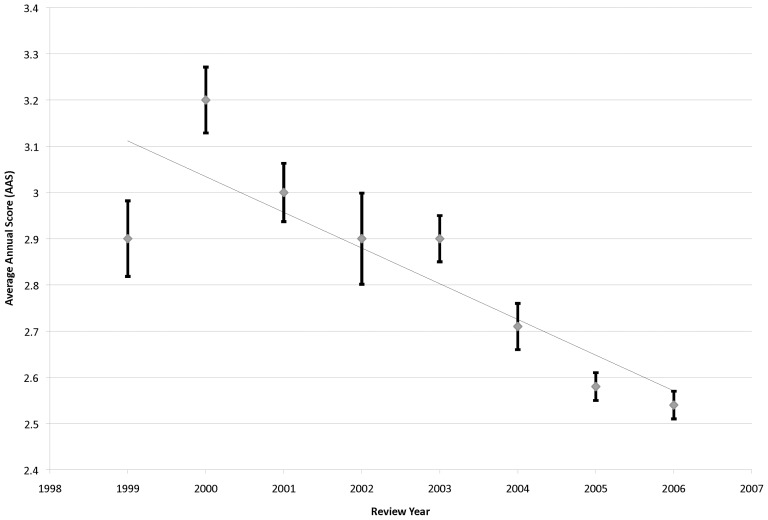
Average Annual Score (AAS) Over Time. Average application score of all applications submitted in a given review year (1999–2006) were plotted against time and fit to a liner regression. Error bars represent the standard error of the mean.

Application scores, averaged for each year (average annual score; AAS), were shown to improve linearly from 1999 to 2006 (**Figure 4**), and were found to be well correlated with time (R^2^ = 0.74, p = 0.006). In addition, both funded and unfunded applications showed an improvement in AAS over time (**Figure S3**), although they remain separated by a substantial gap in score (average 1.2±0.1). As this score trend is seen in both funded and unfunded groups, this suggests that the quality of all submitted applications improved over time. This may have been driven by the overall number of submitted applications (N_s_), which also increased with time and which correlate well with AAS (**Figure S4**, R^2^ = 0.67, p = 0.013).

**Figure 5:**
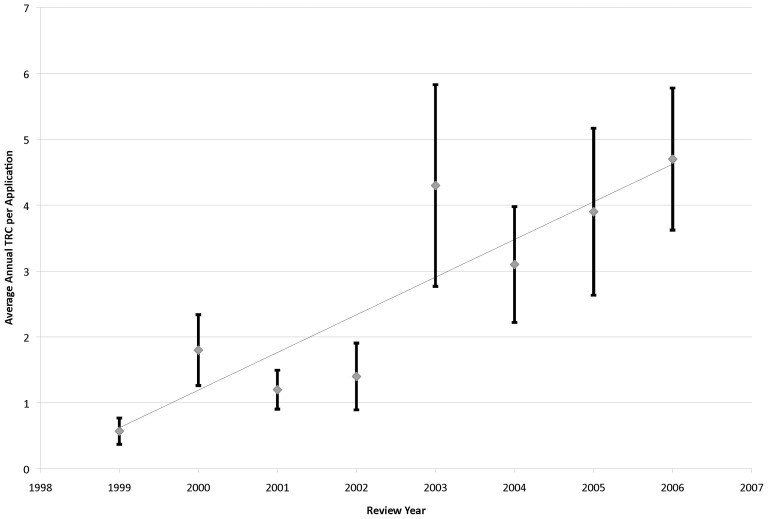
Average Annual TRC Level Over Time. Average application TRC level was determined for all funded applications for each review year (1999–2006), plotted and fit with linear regression. Error bars represent the standard error of the mean.

In an effort to observe temporal changes in citation impact, the average annual TRC over time was plotted in **Figure 5**. A high degree of linear correlation was noted (R^2^ = 0.79, p = 0.003), with average TRC increasing with time with a slope of 0.57±0.12 (p = 0.003). However, the variance was found to increase exponentially (R^2^ = 0.83, p = 0.002; **Figure S5**). A one-way ANOVA of TRC data was conducted over all years, which indicated that these differences over time are statistically significant (F[7,218] = 2.39; p = 0.02).

**Figure 6:**
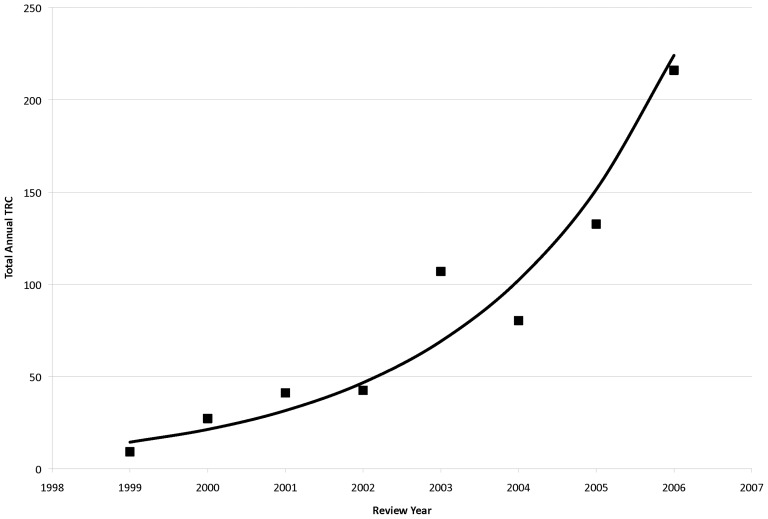
Total Annual TRC Versus Time. Summation of all TRC per year plotted against time and then fit to an exponential function.

**Figure 7:**
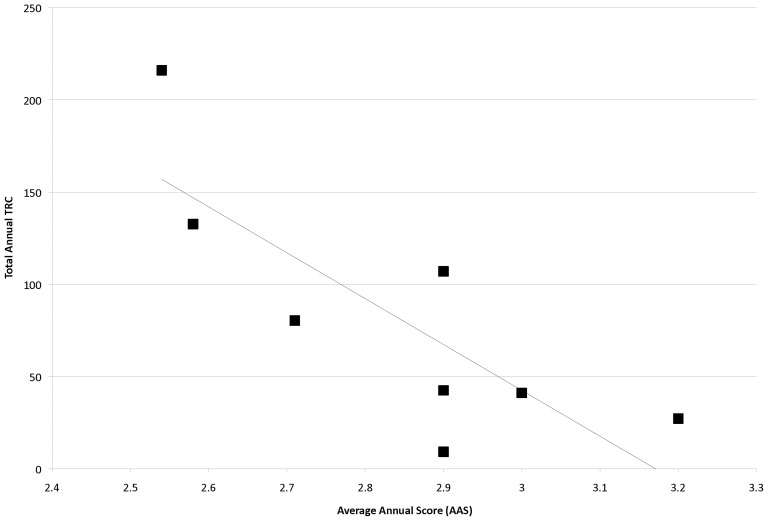
Total Annual TRC Versus AAS. Total annual TRC values were plotted against AAS of submitted applications and then fit to a linear function.

**Figure 8:**
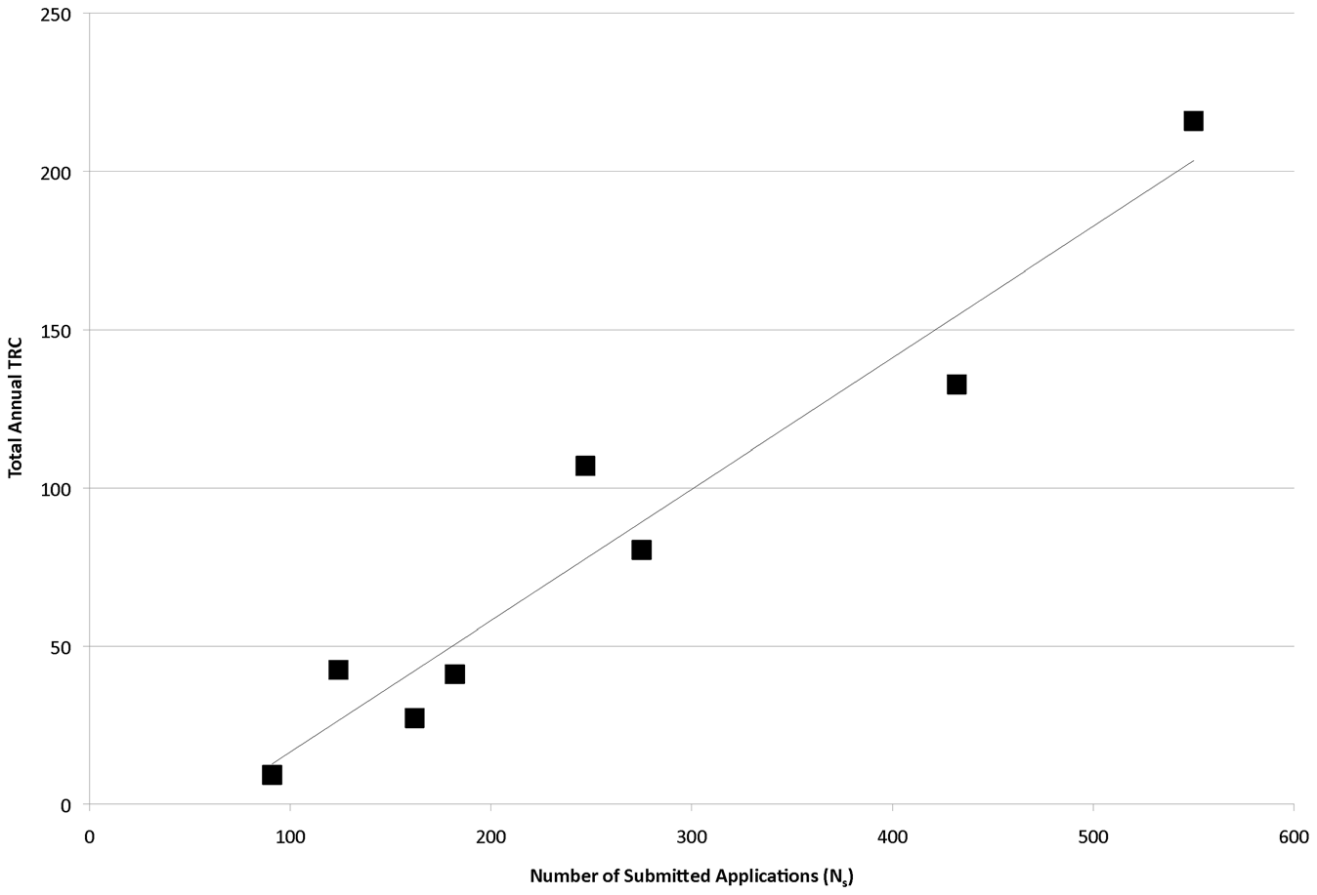
Total Annual TRC Level Versus Number of Submitted (N_s_) Applications per Year. Total annual TRC values were plotted against the corresponding total number of applications submitted for each year and fit to a linear function.

When the total annual TRC (summing up all TRCs from all funded projects from that funding year) is plotted over time, an exponential relationship is noted with a very high degree of correlation (R^2^ = 0.91, p<0.001), with more than a 5-fold increase in annual TRC from 2001 to 2006, all with a relatively level total programmatic budget of roughly $40 million per year (**Figure 6**). This correlation of total annual TRC with time is at least partly due to score, as a linear fit of average annual TRC values versus AAS yielded a slope of −248.7±75.5 (p = 0.010) and a moderate coefficient of determination (R^2^ = 0.64, p = 0.010), with better scores yielding higher TRC values (**Figure 7**). As noted above, N_s_ may be driving temporal improvements in AAS and, therefore, application quality. In fact, there is a high degree of correlation between N_s_ and total annual TRC (**Figure 8**; R^2^ = 0.93, p<0.001), suggesting that larger submitted application pools may lead to increased TRC output.

Total annual TRC values were also found to moderately correlate with the number of applications funded (N_f_) per year (R^2^ = 0.64, p = 0.017); total annual TRC linearly increasing with increasing N_f_ with a slope of 5.4±1.64 (p = 0.017; **Figure S6**). However, total annual TRC was not correlated with total annual programmatic budget (R^2^ = 0.17, p = 0.32). Taken together, this suggests that as submitted applications improve in quality over time and as a larger portfolio of applications is funded, the bibliometric impact of the program overall increases. However, a Lorenz curve analysis of the distribution of TRC for all years over all projects reveals that 30% of all funded grants contribute 89% of the TRC generated by PrX funding, suggesting that there is significant distributional inequality in the impact of funded projects within a given portfolio (**Figure S7**). Overall, these results suggest that N_f_, N_s_ and AAS are correlated with total annual TRC, independent of annual budget and despite the fact that the average budget per project decreased in 2005–2006 when the total annual TRC was the highest.

## Discussion and Conclusions

Significant variability was found in research impact, although much of this was expected, as the funding of research applications is an inherently risk-associated venture. The success of an individual project is dependent on many factors, including external scientific, administrative and personal aspects beyond what can be included or predicted via a research plan. However, our analysis revealed that there was a moderate correlation between peer review scores and citation impact levels (as measured by TRC). Additionally, AAS (both funded and unfunded) improved concomitantly with improvements in average annual TRC levels over time and total annual TRC was found to be well correlated with AAS. Taken together, these data reveal that average peer review scores do have considerable predictive ability with regard to these measures of citation impact, but also considerable variability. However, this correlation is likely under-estimated due to the lack of TRC data related to unfunded applications.

Our findings are in contrast with recent publications from NIH, which indicate little to no correlation between citation levels and peer review scores [14], [15]. One potential difference is the use of standing panels by the NIH versus ad-hoc panels tailored to meet the scientific scope of the submissions by PrX. Another potential difference is that PrX has no resubmission process, which means that all applications are reviewed as new; at the NIH, resubmissions were encouraged during the time period studied. It is possible that score improvements in revised applications are largely based on an applicant's response to reviewers' suggestions, potentially masking the initial—and perhaps more accurate—assessment of the applicant and the overall hypothesis. A third difference is the more permissive funding strategy used in PrX; some funded PrX applications would likely not have been funded under the NIH process, which tends to not fund applications below a certain priority score cut off. The PrX funding strategy allows for exploration further down the scoring scale. Furthermore, more variability was seen in the NHLBI output data than in the PrX data, minimizing the predictive ability of review scores. However, it should be noted that the NHLBI output was focused on a discrete topic area of cardiology, which has a high average citation rate, while PrX output represented a range of topic areas. Clearly, more exploration is needed of the reasons behind these scores to truly understand their basis and any potential to predict the outcome of a project.

If peer review scores have some ability to predict research impact over time, it may be that increases in N_s_ drive the improvements seen in AAS over time for both funded and unfunded applications. It is likely there is a proportional increase in the number of applications with outstanding potential impact as the pool of submitted applications increases, thus giving the funding agency an increasingly improved pool of funding options each year. While conducting many of these peer reviews for both federal and non-federal programs, AIBS has observed similar trends in AAS, not only in extramural funding programs, but also in the review of intramural programs as well, such as the DoD Military Infectious Diseases Research Program (MIDRP) [20]. We are currently exploring the effects of N_s_ on these trends. It should be noted that we do not attribute the PrX improvement in AAS to any kind of reviewer learning (i.e. score creep), as in each year at least 50% of the topic areas are new, as are 80–90% of the reviewers. There are likely multiple reasons for the increased N_s_ over time. Perhaps increased knowledge of the PrX funding opportunity over time by the wider scientific community led to increases in N_s_. In addition, NIH funding rates during this time period decreased, which may have pushed investigators to look for alternative sources of funding.

Concomitantly with the increase in N_s_ over time, the N_f_ per year also increased. While increasing N_f_ is also correlated with increasing total annual TRC, there is an unequal distribution of TRC contribution across projects. Thus, it is likely that increasing the diversity of ideas funded by PrX improves the overall return of this investment by increasing the chances of funding a project in the “heavy tail” portion of the output distribution. This is somewhat analogous to the predictions of the modern portfolio theory of economics and some strategies currently implemented by large funding agencies [21], [22]. Thus, an increase in N_f_ perhaps yields a decrease in portfolio risk. In addition, more applications are being funded, so the cumulative TRC for a given funding year increases. Both the increase in application quality and the decrease in portfolio risk, yield an increase in program impact over time. Most importantly, the total annual funding budget from 2001 to 2006 remained relatively stable, but there was more than a 5-fold increase in total annual TRC over that same time period, implying that the most effective strategy for managing a portfolio of funded applications is to fund more applications at a lower amount per project [23]. This is in line with our observations (and those of others) that budget and bibliometric impact are not well linked, and could have important implications for how research dollars should be allocated [18], [19]. However, this strategy must be weighed against the need for portfolio balance and the fact that some areas of research are inherently more expensive than others [24].

Citations resulting from a funded application are a very limited measure of scientific impact, and a more elaborate panel of bibliometric and non-bibliometric measures will be needed to obtain a more accurate sense of how well peer review scores predict scientific impact, particularly for unfunded applications [25]–[30]. Whatever the measure(s), there is a great need for prospective validation studies of application peer review processes in order to provide a much more robust test to determine what conditions result in the most efficient and accurate peer review [9]. With the PrX program, we observed a correlation between peer review scores and bibliometric impact, which potentially can be utilized as a testing ground for such validation studies, although it is clear more retrospective data need to be gathered before a testable peer review model system, accounting for the full scoring range, can be developed.

Moreover, there is a need for funding agencies to develop a common strategy to identify and collect key metrics both during funding and after it ends. Some efforts are already underway on this front with the STAR METRICS program under the auspices of the White House Office of Science and Technology Policy [31]. Funded institutions should be required to the extent possible as a conditional term of award to provide information to funding agencies for several years after the usual “Final Progress Report” is submitted. Only through using similar metrics and comparing programs directly can the scientific community start to understand and document the successes and failures of research funding and peer review. These types of data are scarce, yet they are crucial for making the best informed research funding decisions to utilize monies in the most impactful and equitable way possible.

## Supporting Information

Figure S1 **TRC Variance Versus Application Score.** TRC was calculated for individual funded applications (1999–2006). Applications were then grouped by identical review score and then averaged. The variance of these TRC values was plotted for the 21 scoring groups (n ranges from 1 to 30, depending on group).
(TIFF)

Figure S2 **Academic Status Versus Average TRC.** Applicants were placed into one of three categories of academic rank: Junior, Mid-level or Senior academic rank groups. Average TRC values for all three of these groups were calculated. Error bars represent the standard error of the mean.
(TIFF)

Figure S3 **AAS Versus Time (Funded and Unfunded).** Average application score of funded and unfunded applications submitted in a given review year (1999–2006) were plotted against time. Error bars represent the standard error of the mean.
(TIFF)

Figure S4 **AAS Versus N_s_ per Year.** The AAS was plotted against the corresponding total number of applications submitted for each year and fit to a linear function. Error bars represent the standard error of the mean.
(TIFF)

Figure S5 **Average Annual TRC Variance Versus Time.** TRC variance was determined for funded applications of each review year (1999–2006), plotted and fit with an exponential regression.
(TIFF)

Figure S6 **Total Annual TRC Versus Number of Funded (N_f_) Applications.** Total annual TRC values were plotted against the number of funded applications per year and then fit to a linear function.
(TIFF)

Figure S7 **Lorenz Curve of TRC Distribution Across Grants.** The cumulative percentile contribution of TRC is plotted against the cumulative percentile of funded projects for all years and all funded projects (1999–2006).
(TIFF)

File S1 **Anonymized Source Data Files.** Anonymized publication, citation, scoring and budget data for each year of funding have been compiled as excel files (compressed as FileS1.zip).
(ZIP)

## References

[1] Cole S, Cole JR, Simon GA (1981) Chance and consensus in peer review. Science 214 (4523): 881–886. 7302566 10.1126/science.7302566

[2] Mervis J (2013) Proposed change in awarding grants at NSF spurs partisan sniping. Science 340(6133): 670. 23661731 10.1126/science.340.6133.670

[3] Berg J (2013) On deck chairs and life boats. ASBMB Today 12: 3–6.

[4] Graves N, Barnett AG, Clarke P (2011) Funding grant proposals for scientific research: retrospective analysis of scores by members of grant review panel. BMJ 343: d4797. 21951756 10.1136/bmj.d4797 PMC3181233

[5] Mayo NE, Brophy J, Goldberg MS, Klein MB, Miller S, et al (2006) Peering at peer review revealed high degree of chance associated with funding of grant applications. J Clin Epidem 59(8): 842–8. 10.1016/j.jclinepi.2005.12.007 16828678

[6] Wood F, Wessely S (2003) Peer review of grant applications: a systematic review. In Peer Review in Health Sciences (ed Godlee, Jefferson) BMJ Publications, London 14–31.

[7] Jackson JL, Srinivasan M, Rea J, Fletcher KE, Kravitz RL (2011) The validity of peer review in a general medicine journal. PLoS ONE 6(7). 10.1371/journal.pone.0022475 PMC3143147 21799867

[8] Van Raan AFJ (2006) Comparison of the Hirsch-index with standard bibliometric indicators and with peer judgement for 147 chemistry research groups. Scientometrics 67(3): 491–502.

[9] Demicheli V, Di Pietrantonj C (2007) Peer review for improving the quality of grant applications. Cochrane Database of Systematic Reviews Library John Wiley and Sons, LTD 2: MR000003. 10.1002/14651858.MR000003.pub2 PMC8973940 17443627

[10] Claveria LE, Guallar E, Cami J, Conde J, Pastor R, et al (2000) Does peer review predict the performance of research projects in health sciences? Scientometrics 47: 11–23.

[11] Bornmann L, Daniel HD (2004) Reliability, fairness and predictive validity of committee peer review. BIF Futura 19: 7–19.

[12] Fang D, Meyer RE (2003) Effect of two Howard Hughes Medical Institute research training programs for medical students on the likelihood of pursuing research careers. Academic Medicine 78(12): 1271–1280. 14660432 10.1097/00001888-200312000-00017

[13] Escobar-Alvarez SN, Myers ER (2013) The Doris Duke clinical scientist development award: implications for early-career physician scientists. Academic Medicine 88(11): 1–7. 24072110 10.1097/ACM.0b013e3182a7a38e

[14] Berg JM (2013) Scientific approaches to science policy. Molecular Biology of the Cell 24: 3273–3274. 24174459 10.1091/mbc.E13-07-0400 PMC3814158

[15] Danthi N, Wu CO, Shi P, Lauer MS (2014) Percentile ranking and citation impact of a large cohort of NHLBI-funded cardiovascular R01 grants. Circ. Res 114(4): 600–6. 24406983 10.1161/CIRCRESAHA.114.302656 PMC3959724

[16] Gallo SA, Carpenter AS, Glisson SR (2013) Teleconference versus face-to-face scientific peer review of grant application: effects on review outcomes. PLoS ONE 8(8). 10.1371/journal.pone.0071693 PMC3740535 23951223

[17] Citation Averages, 2000–2010, By Fields and Years http://www.timeshighereducation.co.uk/415643.article (2011) Accessed 2013 March 10.

[18] Fortin JM, Currie DJ (2013) Big science vs. little science: how scientific impact scales with funding. PLoS ONE 8(6).. 10.1371/journal.pone.0065263 PMC3686789 23840323

[19] Wadman M (2010) Study says middle-sized labs do best. Nature 468: 356–357. 21085145 10.1038/468356a

[20] Glenn JF (2010) Uses and needs for peer review in army medical research. Technology and Innovation 12: 241–247.

[21] Markowitz HM (1991) Foundations of portfolio theory. The Journal of Finance 46: 469–477.

[22] Galis ZS, Hoots WK, Kiley JP, Lauer MS (2012) On the value of portfolio diversity in heart, lung, and blood research. Am J Respir Crit Care Med 186(7): 575–578. 23027846 10.1164/rccm.201208-1437ED PMC3480520

[23] Ioannidis JPA (2011) More time for research: fund people not projects. Nature 477(7366): 529–531. 21956312 10.1038/477529a

[24] Lauer MS (2014) Personal reflections on big science, small science, or the right mix. Circ. Res. 114: 1080–1082. 10.1161/CIRCRESAHA.114.303627 PMC4006109 24677234

[25] Van Raan AFJ (2004) Measuring science. In: Handbook of Quantitative Science and Technology Research (eds, HF Moed et al.), Kluwer Academic Publishers, Dordrecht, Netherlands. 19–50.

[26] Braun T, Bergstrom CT, Frey BS, Osterloh M, West JD, et al (2010) How to improve the use of metrics. Nature 465(17): 870–872.20559368

[27] Cabezas-Clavijo A, Robinson-Garcia N, Escabias M, Jiménez-Contreras E (2013) Reviewers' ratings and bibliometric indicators: hand in hand when assessing over research proposals? PLoS ONE 8(6).. 10.1371/journal.pone.0068258 PMC3695904 23840840

[28] Van Noorden R (2010) Metrics: a profusion of measures. Nature 465(7300): 864–866. 20559362 10.1038/465864a

[29] de Winter JCF, Zadpoor AA, Dodou D (2014) The expansion of google scholar versus web of science: a longitudinal study. Scientometrics 98(2): 1547–1565.

[30] Dwan K, Altman DG, Arnaiz JA, Bloom J, Chan AW, et al (2008) Systematic review of the empirical evidence of study publication bias and outcome reporting bias. PLoS ONE 3(8).. 10.1371/journal.pone.0003081 PMC2518111 18769481

[31] Lane J (2010) Let's make science metrics more scientific Nature. 464(25): 488–489. 10.1038/464488a 20336116

