# Supplementary figures and images for: The Validation of Peer Review through Research Impact Measures and the Implications for Funding Strategies
Source: PLoS One. 2014 Sep 3;9(9):e106474. doi: 10.1371/journal.pone.0106474 (PMC4153641; doi:10.1371/journal.pone.0106474)

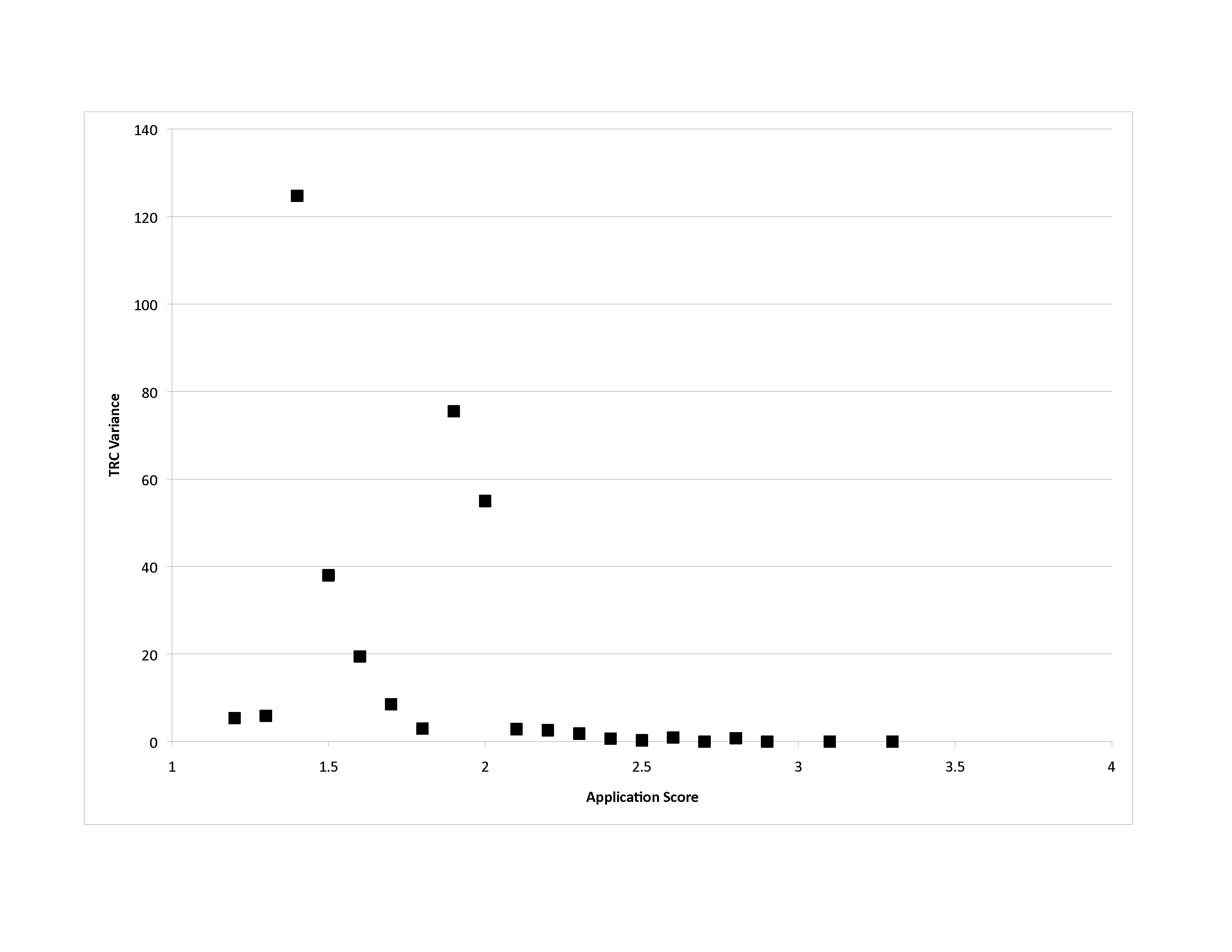

Supplement: Figure S1 — TRC Variance Versus Application Score. TRC was calculated for individual funded applications (1999–2006). Applications were then grouped by identical review score and then averaged. The variance of these TRC values was plotted for the 21 scoring groups (n ranges from 1 to 30, depending on group). (TIFF) [file pone.0106474.s001.tiff]

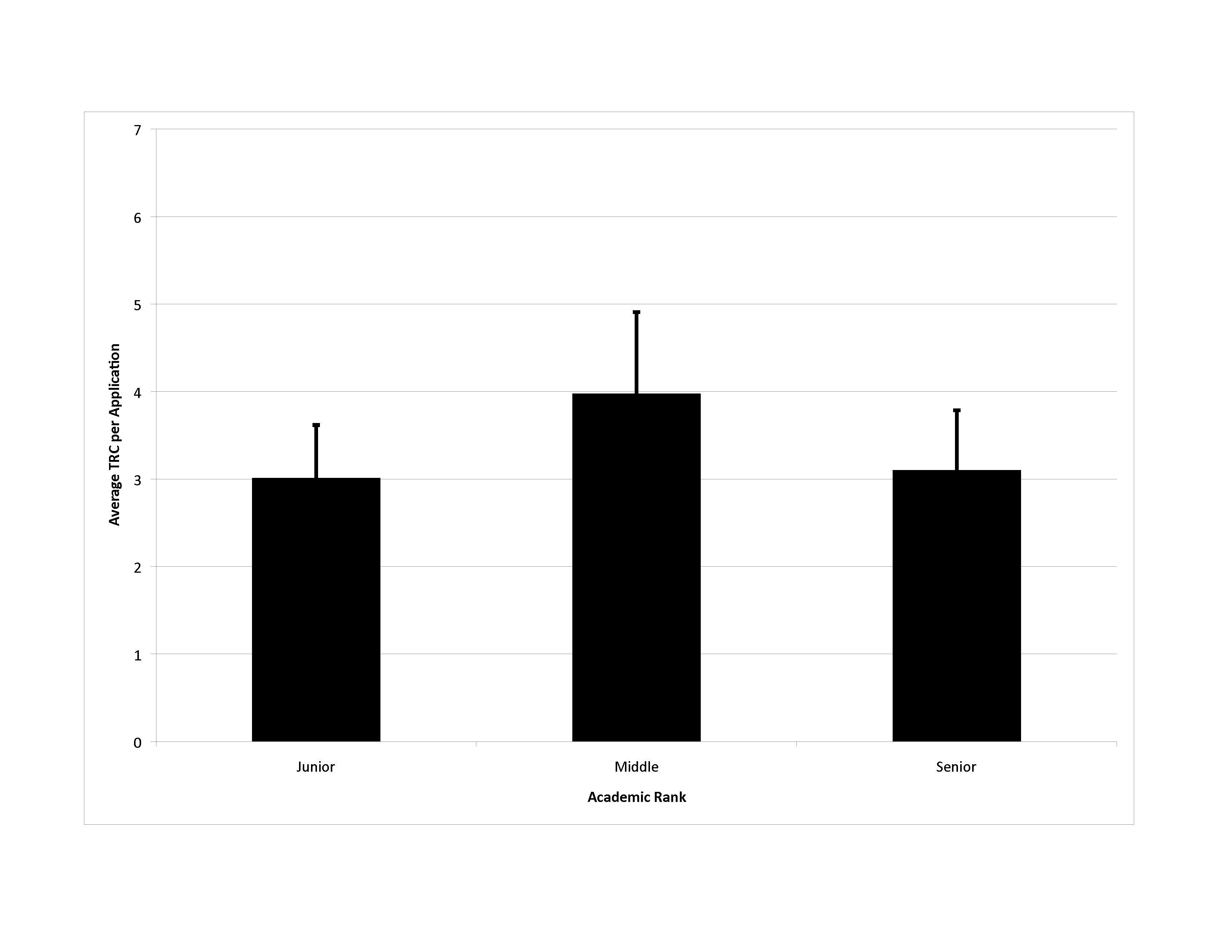

Supplement: Figure S2 — Academic Status Versus Average TRC. Applicants were placed into one of three categories of academic rank: Junior, Mid-level or Senior academic rank groups. Average TRC values for all three of these groups were calculated. Error bars represent the standard error of the mean. (TIFF) [file pone.0106474.s002.tiff]

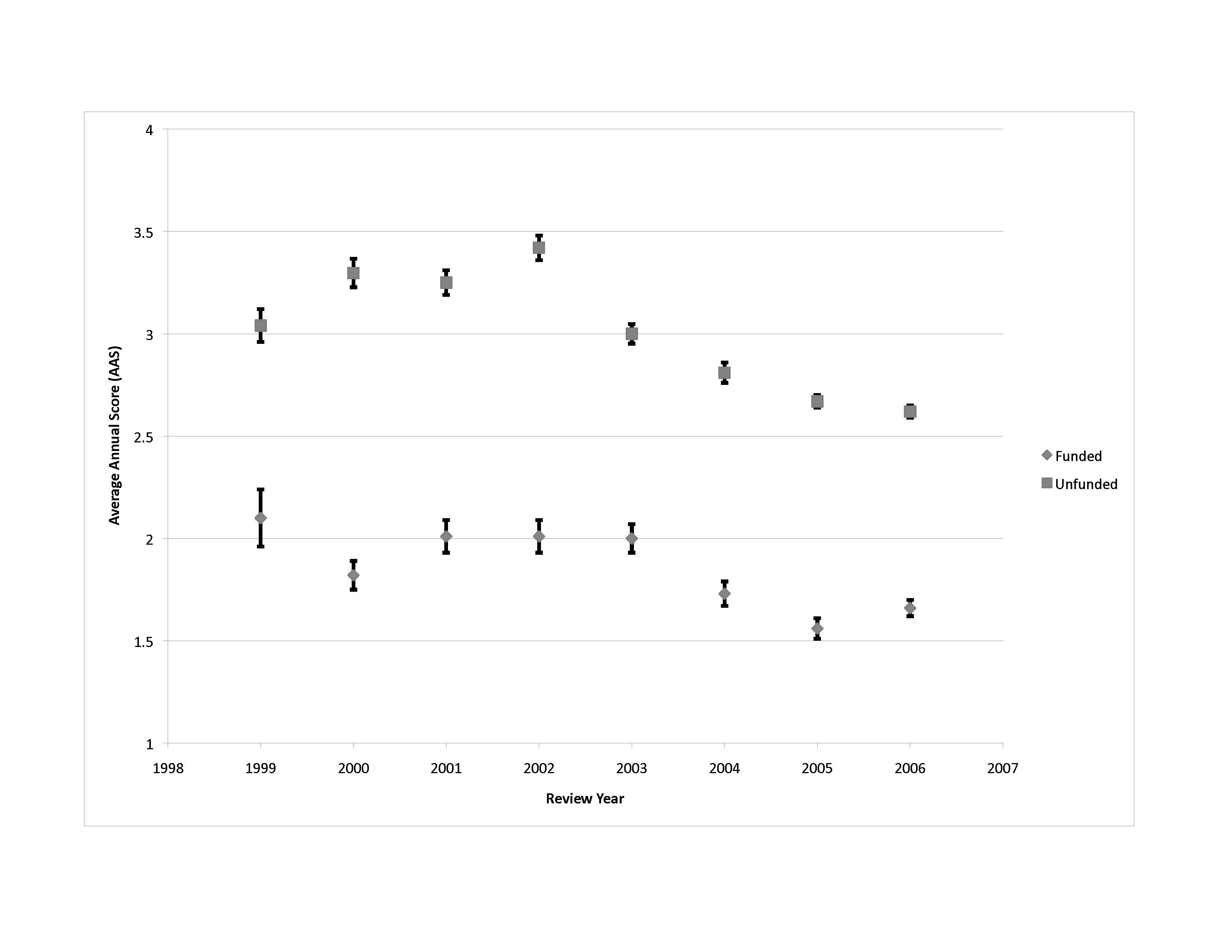

Supplement: Figure S3 — AAS Versus Time (Funded and Unfunded). Average application score of funded and unfunded applications submitted in a given review year (1999–2006) were plotted against time. Error bars represent the standard error of the mean. (TIFF) [file pone.0106474.s003.tiff]

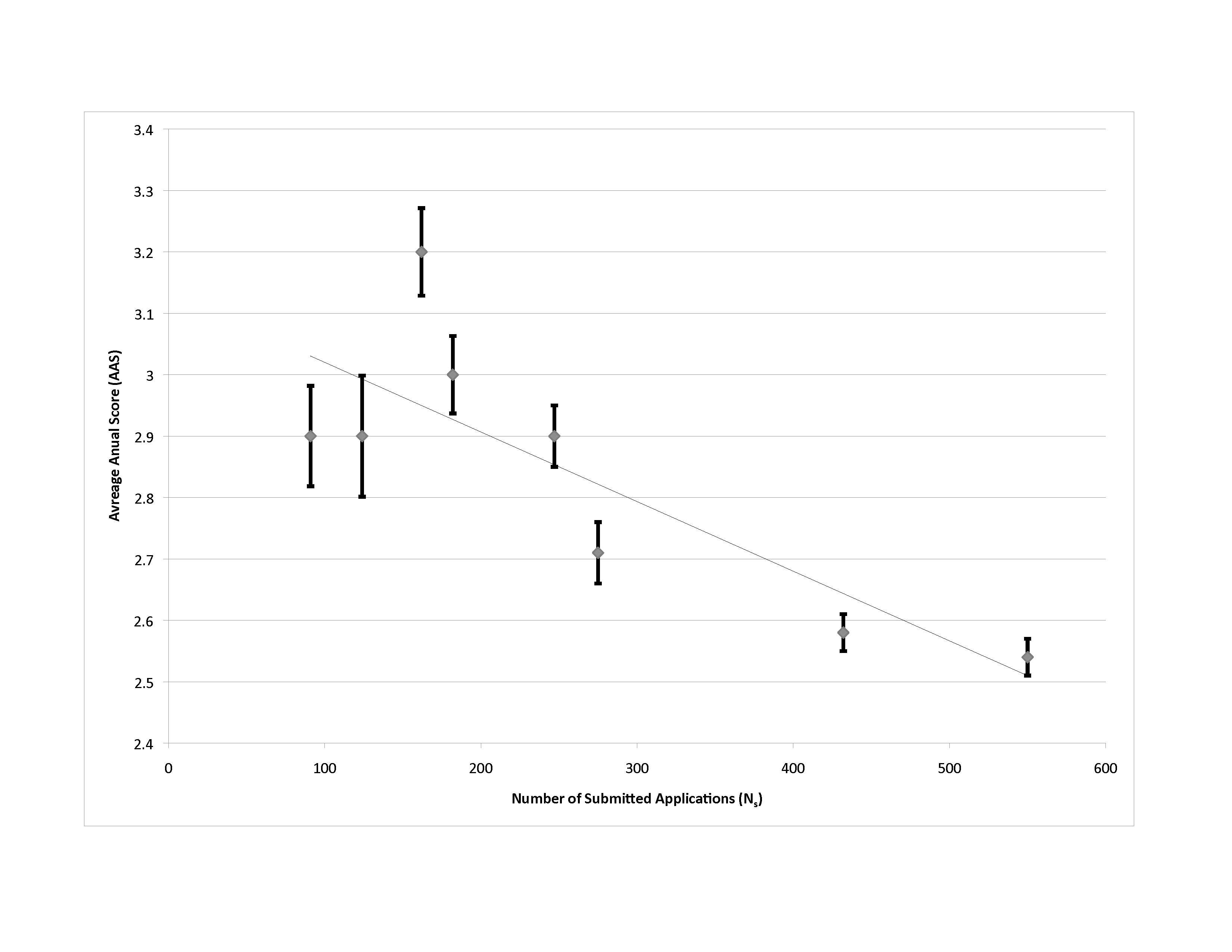

Supplement: Figure S4 — AAS Versus Ns per Year. The AAS was plotted against the corresponding total number of applications submitted for each year and fit to a linear function. Error bars represent the standard error of the mean. (TIFF) [file pone.0106474.s004.tiff]

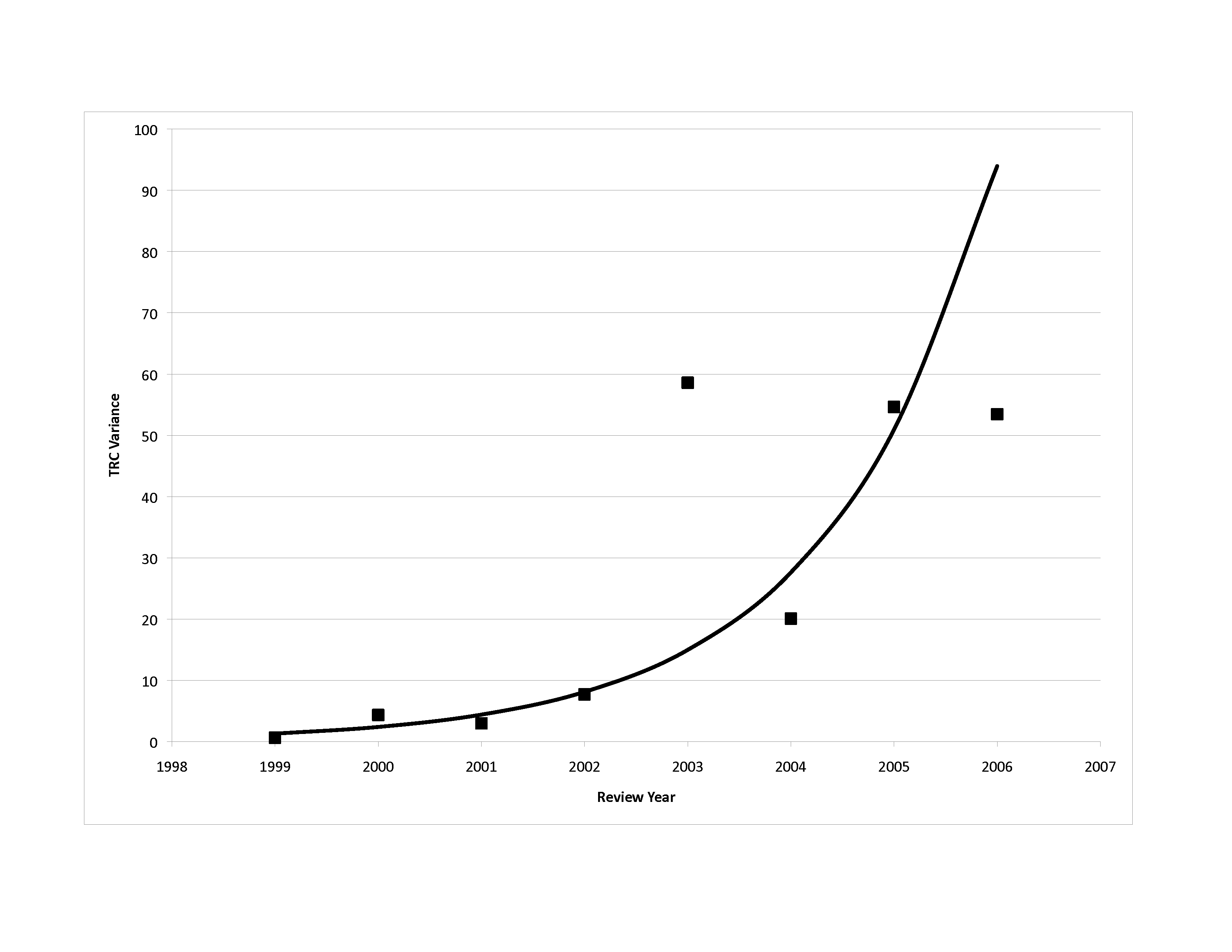

Supplement: Figure S5 — Average Annual TRC Variance Versus Time. TRC variance was determined for funded applications of each review year (1999–2006), plotted and fit with an exponential regression. (TIFF) [file pone.0106474.s005.tiff]

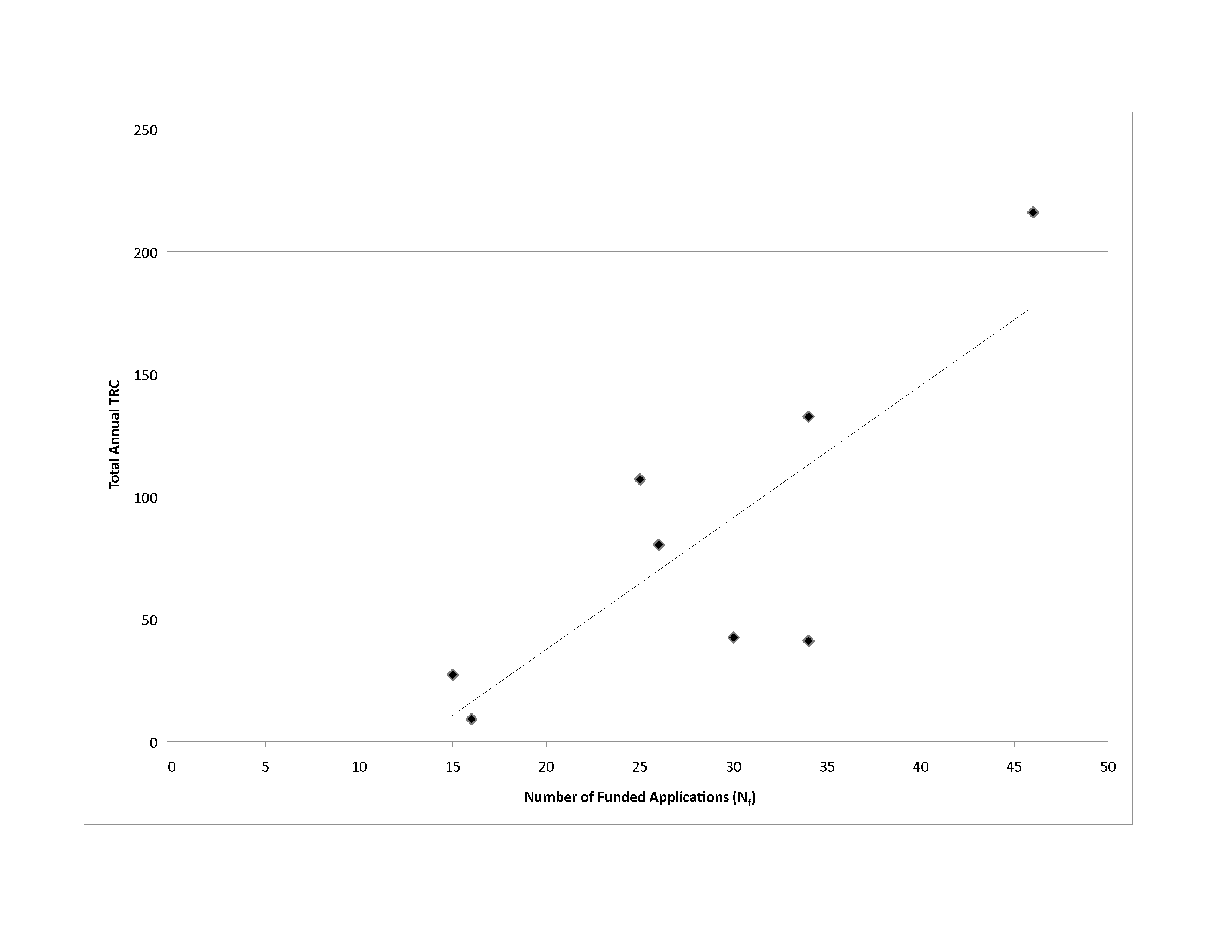

Supplement: Figure S6 — Total Annual TRC Versus Number of Funded (Nf) Applications. Total annual TRC values were plotted against the number of funded applications per year and then fit to a linear function. (TIFF) [file pone.0106474.s006.tiff]

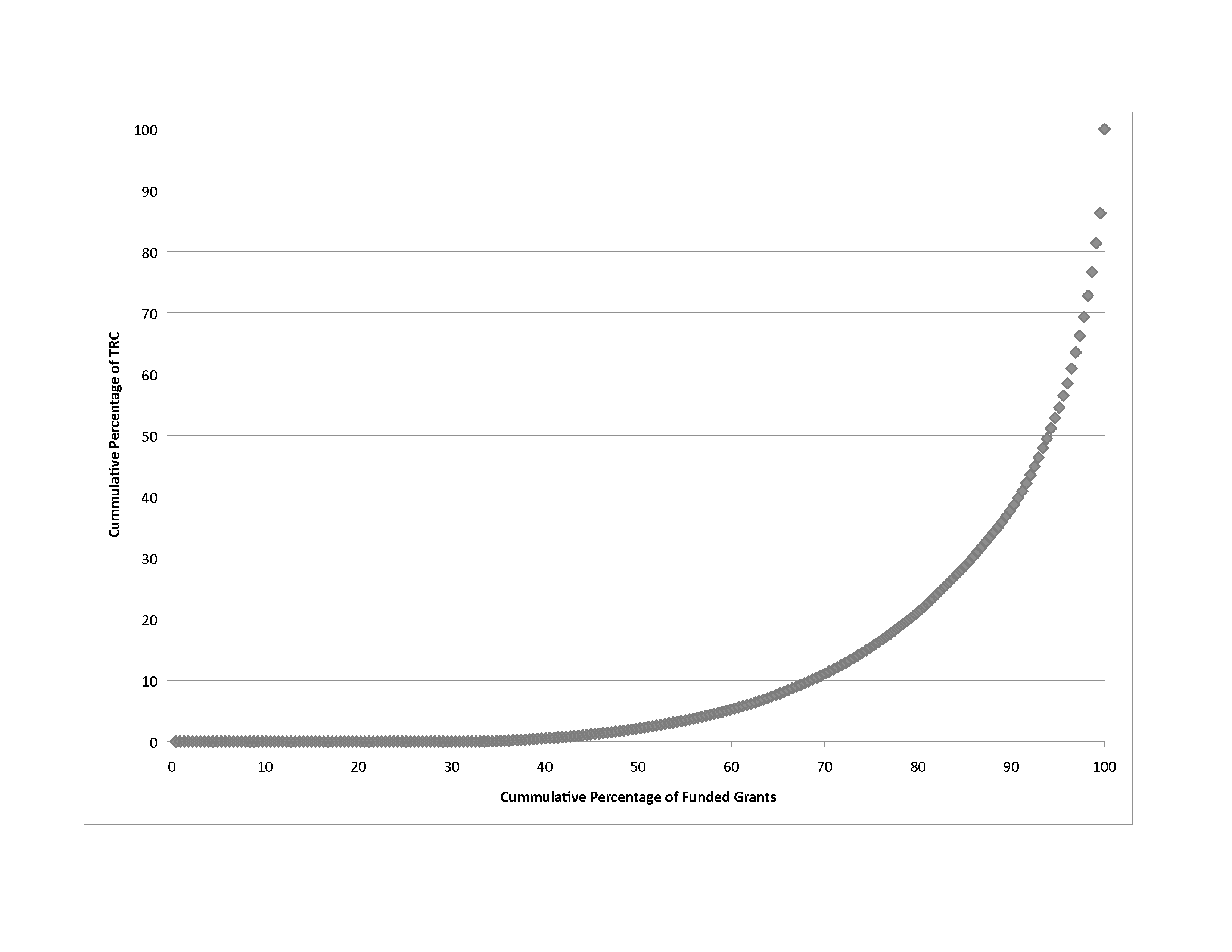

Supplement: Figure S7 — Lorenz Curve of TRC Distribution Across Grants. The cumulative percentile contribution of TRC is plotted against the cumulative percentile of funded projects for all years and all funded projects (1999–2006). (TIFF) [file pone.0106474.s007.tiff]
